# Relationships between Changes in Hematological Adaptations and Exercise Capacity in Olympic Rowers after a Period of Reduced Training Loads

**DOI:** 10.5114/jhk/159463

**Published:** 2023-01-20

**Authors:** Dariusz Sitkowski, Andrzej Klusiewicz, Andrzej Pokrywka, Wojciech Jankowski, Jadwiga Malczewska-Lenczowska

**Affiliations:** 1 Department of Physiology, Institute of Sport – National Research Institute, Warsaw, Poland.; 2 Department of Biochemistry and Pharmacogenomics, Faculty of Pharmacy, Medical University of Warsaw, Warsaw, Poland.; 3 Polish Federation of Rowing Associations, Warsaw, Poland.; 4 Department of Physiology of Nutrition and Dietetics, Institute of Sport – National Research Institute, Warsaw, Poland.

**Keywords:** endurance athletes, graded exercise test, total hemoglobin mass, plasma volume, blood volume

## Abstract

Endurance performance is positively associated with hematological adaptations; therefore, high total hemoglobin mass and intravascular volumes are commonly observed in high-level endurance athletes. However, it is still unclear whether the fluctuations in exercise capacity that typically occur in endurance athletes during the annual training cycle are directly associated with changes in hematological adaptations, which appear to be relatively stable during this time. To better understand this issue, a study was conducted with 10 Olympic rowers, who followed the same training program. Athletes underwent laboratory testing in the competitive and the general preparation phase of an annual training cycle (a 34% reduction in training volume). This included a graded exercise test on a rowing ergometer (GXT) and blood measurements of hemoglobin concentration (Hb), total hemoglobin mass (tHb-mass), plasma volume (PV), and blood volume (BV). Decreases in maximal values of power relative to body mass (p = 0.028), lactate concentration (p = 0.005), and heart rate (p = 0.017) in the GXT were registered. At the same time, absolute (p = 0.017) and relative (p = 0.005) PV decreased. Changes in PV (rS = 0.842, p = 0.002) and BV (rS = 0.818, p = 0.004), but not in tHb-mass (rS = 0.588, p = 0.074) and Hb (rS = −0.188, p = 0.602), were significantly correlated with changes in maximal power in the GXT. Our results indicate a close relationship between changes in intravascular volumes and maximal exercise capacity after a period of reduced training loads in elite endurance athletes.

## Introduction

One of the most important determinants of maximal oxygen uptake (VO_2max_) and endurance performance is oxygen delivery to the exercising muscles (Bassett and Howley, 2000). In this regard, elite endurance athletes exhibit much higher total hemoglobin mass (tHb-mass) and intravascular volumes than untrained individuals, non-endurance athletes, or even their less successful counterparts (Heinicke et al., 2001; Zelenkova et al., 2019). Whilst tHb-mass and blood volume (BV) in athletes correlate strongly with endurance performance predictors (EPP), such as maximal or submaximal power and VO_2max_ (Heinicke et al., 2001; Treff et al., 2014b), no correlations or only moderate ones between post-training changes (Δ) in tHb-mass and ΔEPP are most frequently reported (Friedmann et al., 2005; Yan et al., 2021). Nevertheless, there are data showing a strong correlation between changes in endurance running performance and ΔtHb-mass after altitude training (Hauser et al., 2016).

Although the effects of different training methods on changes in hematological adaptations have been widely studied, the effect of reduced training loads on such adaptations is still poorly understood, especially in elite athletes. As previously demonstrated, tHb-mass may decrease by 15–19% following prolonged reduction in training loads or immobilization due to injury (Gough et al., 2013; Kjellberg et al., 1949). Similarly, a large reduction in tHb-mass (28%) was reported in an elite rower suffering from gastrointestinal blood loss, even though training volume was maintained (Treff et al., 2014a). However, a 100% decrease in the 6-week training load of healthy athletes was expected to lower tHb-mass by ~11%, based on estimates from a regression model (Garvican et al., 2010). Others have also observed a decrease in tHb-mass (of ~3%) after 30 days of partial detraining (87% reduction in training volume) following an ultra-endurance triathlon race (Eastwood et al., 2012). On the other hand, in competitive endurance athletes, tHb-mass remained relatively stable throughout the annual training cycle despite 25% fluctuations in training volume (Prommer et al., 2008). Likewise, during a shorter observation period (100 days of the competitive season), only 2–3% variations in tHb-mass were reported in both recreationally active men and elite female cyclists (Eastwood et al., 2008; Garvican et al., 2010). Furthermore, the results of a 9-year observation in 511 competitive athletes showed that, despite ending a sports career and/or a marked reduction in training volume, tHb-mass remained unchanged in this cohort (Schmidt and Prommer, 2008). This finding suggests that tHb-mass is relatively stable, depending mainly on genetic predisposition, with only slight training-induced modifications. Therefore, it is postulated that tHb-mass in adolescent athletes may be an important predictor of later national team membership in endurance sports (Wehrlin and Steiner, 2021) and may be a useful variable to detect blood doping (Prommer et al., 2008).

Given this relative stability in tHb-mass in healthy and regularly trained athletes, coupled with substantial variation in EPP values throughout the annual training cycle (Anderson et al., 2006; Mikulic, 2012), an important question arises as to how it relates to oxygen delivery to exercising muscles as the main determinant of VO_2max_ and endurance performance (Bassett and Howley, 2000). Since oxygen delivery is a product of arterial oxygen content ((CaO2 = (hemoglobin concentration [Hb] x 1.34 x arterial oxygen saturation [SaO2]) + (0.0031 x arterial oxygen pressure [PaO2])) and cardiac output (CO = stroke volume [SV] x heart rate [HR]), it can also be affected by changes in intravascular volumes. Indeed, ΔBV (but not ΔHb) seems to be the main regulator of oxygen transport to the exercising muscles in physiological non-anemic conditions (Schmidt and Prommer, 2008). However, fluctuations in PV due to acute physical strain caused by training and competition, which are often conducted in extreme environmental conditions (e.g. heat, cold, and altitude), can affect BV and Hb (Astolfi et al., 2021; Sawka et al., 2000).

Given these discrepancies, our study’s aim was to investigate the relationship between changes in hematological adaptations and changes in exercise capacity in male Olympic rowers after a period of reduced training loads. We hypothesized that training-induced changes in maximal and submaximal power in a graded exercise test on a rowing ergometer would be associated with changes in intravascular volumes.

## Methods

### Participants

Following ethical approval (KEBN-15-15-JM) by a local ethics committee, and in accordance with the Declaration of Helsinki, the current study examined 10 world-class rowers (men’s coxed eight and substitutes; mean age: 26.6 ± 3.3 years; body mass: 97.7 ± 5.4 kg; body height: 198 ± 6 cm; training experience: 11.4 ± 3.3 years; VO_2peak_: 64.2 ± 4.3 ml/kg/min). All study participants were members of the national Olympic team, which included prior Olympic Games finalists and medalists at world and continental championship events. Informed written consent was obtained from all study participants. The current sample size was estimated *a priori* using power analysis software (G*Power; v3.1.9.2), based on an anticipated change in blood volume of 0.53 ± 0.37 l (Eastwood et al., 2012) from baseline (i.e., paired *t-*test, two-tailed), with alpha set to 0.05 and power equal to 0.95.

### Design and Procedures

Athletes were assessed twice during an annual training cycle: (1) a competitive phase (July), and (2) a general preparation phase (January). These two time points represented distinct periods with different competitive goals, that is, approaching the competitive peak for these athletes and at the lowest competitive phase, respectively. Laboratory testing included: body mass (BM) assessment, blood collection, medical examination, a graded exercise test (GXT), and measurements of total hemoglobin mass (tHb-mass) and intravascular volumes (PV and BV).

### Training

During each observation period and between testing occasions, rowers, as one crew, underwent (at altitudes ranging from near sea level to 900 m) the same prescribed training program. Substitutes, who were changed occasionally during the competitive phase, trained on water and competed in this period in the coxless pairs event. Training volume (time) and intensity (at the discretion of the coach) were strictly controlled. During rowing training on the water or on the ergometer, the intensity was assessed by measuring boat velocity (Nielsen-Kellerman SpeedCoach) or mechanical power (Concept2 measurement system), respectively. Additionally, during selected training sessions (including running, cycling, and cross-country skiing), heart rate measurements and random measurements of lactate concentration in capillary blood were performed. Based on the daily data collected, training intensity was classified into three zones: extensive aerobic endurance (Z1), intensive aerobic endurance (Z2), and speed/anaerobic endurance (Z3). Overall characteristics of training conducted during the two-month periods preceding July and January testing are presented in Table 1.

**Table 1:** Volume, percentage contribution, and intensity distribution (Z1/Z2/Z3) of training conducted during 2 months preceding testing.

Variable	Competitive phase (June–July)	General preparation phase (December–January)
Total training volume (min)	7953	5283
On-water (%)	56 (86/12/2)	0
Rowing ergometer (%)	9 (73/24/3)	46 (76/23/1)
Running (%)	7 (100/0/0)	2 (100/0/0)
Cycling (%)	15 (33/67/0)	0
Cross country skiing (%)	0	27 (44/56/0)
Strength/core/functional training (%)	13	25

Z1 – extensive aerobic endurance, Z2 – intensive aerobic endurance, Z3 – speed/anaerobic endurance. The intensity distribution of strength, core and functional training was not rated due to a lack of reliable criteria.

### Measures

#### Body Mass

Body mass was assessed (with accuracy of 50 g) immediately after waking and urination using an electronic scale (Dolphin, CAS Corp., South Korea). Athletes wore only briefs for this assessment.

#### Blood Analysis and Medical Examination

Athletes visited the laboratory in a fasted state between 7:30 and 8:00 a.m. After a 15-min rest in a sitting position, 2 blood samples (2 ml each) were collected from the antecubital veins. Blood morphology was assessed (Advia 120, Bayer, Germany) and ferritin, iron, unsaturated iron-binding capacity (UIBC), C-reactive protein (CRP) (Pentra 400, Horiba Medical, France), and soluble transferrin receptor (sTfR) (ELISA method, Ramco Laboratories, USA) were measured. From the sum of iron and UIBC, total iron-binding capacity (TIBC) was calculated. Blood collection was followed by a medical examination, including screening of medical history, auscultation of the heart and lungs, electrocardiography, and blood pressure measurement, all supervised by a physician.

#### Graded Exercise Test

About 90 min after a light breakfast, the GXT (3-min stages with 30-s rest periods) was performed on a Concept 2/Model D (Morrisville, USA) wind-braked rowing ergometer. Since this study constituted part of a training process supervised by the national rowing federation, all athletes were familiar with the ergometer testing procedures. Initial power and stroke rates in the GXT were set at 170 W and 14 strokes/min, respectively, and increased by 50 W and 2 strokes/min at consecutive stages. The ergometer damper fan was set to positions 4–6. The GXT was stopped when the athlete was unable to maintain the required power output. Immediately after each stage and 3 min after completion of the GXT (La3’), 20 µl capillary blood samples were collected from the earlobe to evaluate lactate (La) concentration (Super GL2, Dr. Müller, Gerätebau GmbH, Germany). Submaximal power values corresponding to the La threshold (at La = 4 mmol/l and determined using the modified Dmax method) were calculated by self-developed software, based on a third-order polynomial curve. Maximal power output (P_max_) was calculated according to the following formula:


$$P_{max}=\;P_{pfin}+\;(t_{fin}*\;(P_{fin}–\;P_{pfin}))\;/\;180$$

where: P_pfin_ = power at the pre-final step, Pfin = power at the final step, tfin = time of sustaining power at the final step. Heart rate (HR) was recorded continuously (S610i HR monitor, Polar Electro Oy, Finland) throughout the exercise; the highest value of HR measured with 15-s averaging was taken as maximal (HR_max_).

#### Total Hemoglobin Mass and Intravascular Volumes

During the afternoon session (at least 3 hours following exercise testing), and after detailed instructions on how to perform a special breathing maneuver, tHb-mass and intravascular volumes were determined using an optimized carbon monoxide rebreathing method (Schmidt and Prommer, 2005). The administered gas mixture consisted of CO (1.0 ml/kg BM) and 99.5% O2 (4 l bag). A CO sensor (Pac 7000, Dräger, Germany) was used to check potential leaks in exhaled air before and after the test. Blood (~105 µl) was sampled from the hyperemized (Finalgon, Boehringer Ingelheim, Germany) earlobe in the following order: just before the test (4 samples), and in the 6^th^ (2 samples) and 8^th^ minutes (3 samples) from the beginning of inhalation of the gas mixture (lasting 2 min). The samples served to determine the percentage value of carboxyhemoglobin (HbCO%) using a CO oximeter (ABL 80 Flex, Radiometer, Denmark). Computer software (Blood Volume Measurements: SpiCO; Blood tec, Bayreuth, Germany) was employed to calculate tHb-mass, BV, and PV. The typical error of tHb-mass measurement, periodically determined in our laboratory based on duplicate measurements, did not exceed 2.0%.

### Statistical Analysis

The results are presented as means with standard deviations (± SD) and expressed in absolute values and relative to BM (rel.). Mean differences between the two time points were tested using the Wilcoxon’s matched-pairs test. Cohen’s *r* effect sizes (*r*ES = z/√N) were also calculated (values for *r*ES of ≤ 0.1, ≤ 0.3, and ≥ 0.5 were considered small, moderate, and large, respectively) (Fritz et al., 2012). Spearman’s rank correlation analysis was used to determine the relationship between percentage changes in maximal and submaximal power in the GXT and changes in intravascular volumes, tHb-mass, and Hb. The level of significance was set at *p* < 0.05. Statistica 13.1 software (Dell Inc., USA) was employed.

## Results

### Phase Differences

Participants did not report any serious health issues during the entire study, and they trained according to the prescribed program. At both time points of observation, the mean values of all indices of iron status and CRP were within reference ranges (Table 2), and none of the athletes were found to be iron deficient.

**Table 2:** Mean values (± SD) of iron status and inflammation indices at the two observation time points.

	*Reference range*	Competitive phase (July)	General preparation phase (January)
Ferritin (μg/l)	*20*–*250*	69 ± 27	70 ± 32
Iron (μgx/dl)	*40*–*155*	106 ± 32	116 ± 38
TIBC (µg/dl)	*270*–*410*	304 ± 25	293 ± 18
sTfR (mg/l)	*2.9–8.3*	4.9 ± 0.8	6.0 ± 1.2
CRP (mg/l)	*0.0–5.0*	0.14 ± 0.15	0.11 ± 0.07

TIBC – total iron-binding capacity, sTfR – soluble transferrin receptor, CRP – C-reactive protein.

At the same time, mean BM increased, while rel.P_max_, La_3_', HR_max_, PV, and rel.PV decreased (Table 3).

**Table 3:** Mean values (± SD) of exercise and hematological variables at the two observation time points, as well as average values of individual percentage changes (95% CI), the significance level of the difference between means, and Cohen’s r effect sizes.

Variable	Competitive phase (July)	General preparation phase (January)	Percentage changes (95% CI)	*p* values	Cohen’s *r*
BM (kg)	97.7 ± 5.4	100.2 ± 6.1	3 (1; 4)	0.022	0.513
*Graded exercise test variables*					
P_max_ (W)	485 ±12	478 ± 24	−2 (−5; 1)	0.285	0.239
rel.P_max_ (W/kg)	4.98 ± 0.23	4.78 ± 0.40	−4 (−7; −1)	0.028	0.490
La_3’_ (mmol/l)	14.2 ± 1.9	11.9 ± 2.3	−16 (−23; −9)	0.005	0.627
HR_max_ (bpm)	193 ± 6	188 ± 9	−2 (−4; 0)	0.017	0.536
PD_maxM_ (W)	374 ± 9	374 ± 18	0 (−4; 4)	0.508	0.148
rel.PD_maxM_ (W/kg)	3.84 ± 0.15	3.75 ± 0.32	−2 (−7; 2)	0.445	0.171
P_AT4_ (W)	383 ± 12	389 ± 22	2 (−2; 5)	0.386	0.194
rel.P_AT4_ (W/kg)	3.92 ± 0.14	3.90 ± 0.33	−1 (−6; 4)	0.878	0.034
*Hematological variables*					
tHb−mass (g)	1367 ± 130	1361 ± 83	0 (−3; 3)	0.959	0.011
rel.tHb−mass (g/kg)	14.0 ± 0.8	13.6 ± 0.5	−2 (−7; 2)	0.193	0.291
Hb (g/dl)	15.5 ± 0.9	15.4 ± 0.9	0 (−2; 2)	0.878	0.066
PV (ml)	5940 ± 773	5678 ± 683	−4 (−8; −1)	0.017	0.536
rel.PV (ml/kg)	61 ± 5	57 ± 5	−7 (−9; −4)	0.005	0.627
BV (ml)	9759 ±1092	9742 ± 886	0 (−3; 3)	0.878	0.034
rel.BV (ml/kg)	100 ± 7	97 ± 6	−2 (−6; 1)	0.139	0.330

BM – body mass, P_max_ – maximal power, rel. – relative to body mass, La_3_' – blood lactate concentration 3 min after completion of the graded exercise test, HR_max_ – the highest heart rate, PDmaxM – power at lactate threshold determined by modified Dmax method, PAT4 – power at lactate threshold with fixed La value of 4 mmol/l, tHb-mass – total hemoglobin mass Hb – hemoglobin concentration, PV – plasma volume, BV – blood volume.

### Correlations

**Figure 1:**
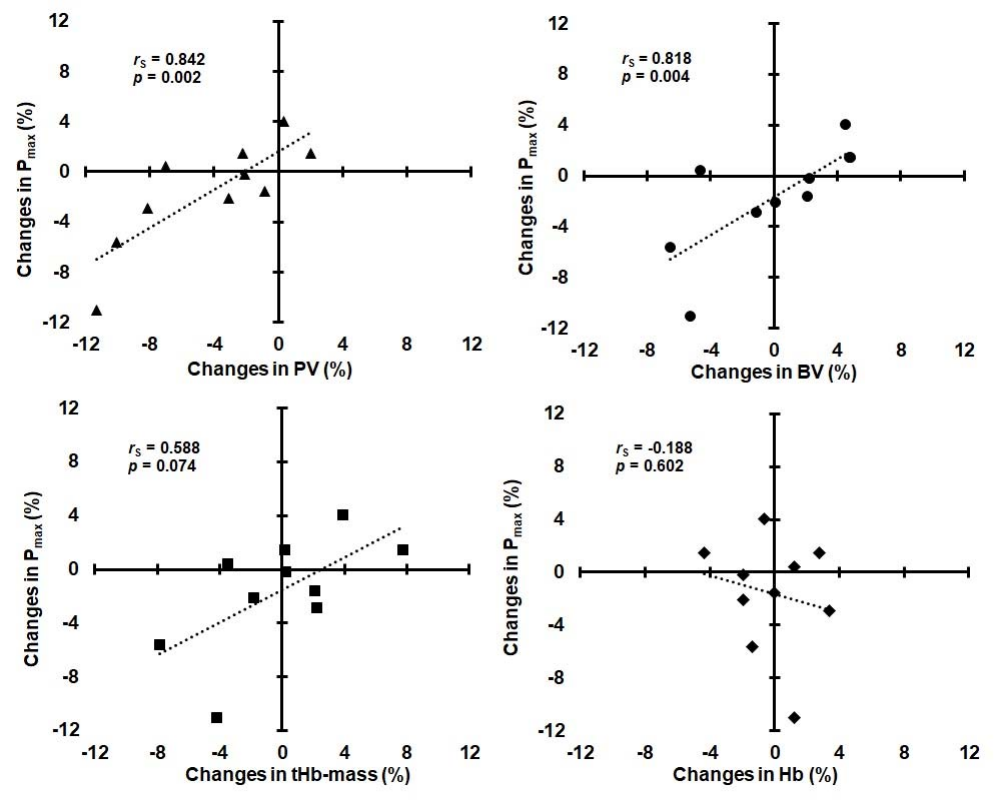
Relationships between changes in maximal power (P_max_) and changes in plasma volume (PV), blood volume (BV), total hemoglobin mass (tHb-mass), and hemoglobin concentration (Hb).

ΔP_max_ was significantly correlated with ΔPV and ΔBV, but not with ΔtHb-mass and ΔHb (Figure 1).

Changes in submaximal power (ΔPDmaxM and ΔPAT4 ) were not significantly correlated with ΔPV, ΔBV, and ΔtHb-mass (*r*S ranged from 0.152 to 0.455, *p* ranged from 0.187 to 0.676), as well as with ΔHb (*r*S = 0.103, *p* = 0.776 and *r*S = 0.304, *p* = 0.393, respectively). ΔPV was not significantly correlated with ΔHb (*r*S = −0.468, *p* = 0.173), whereas ΔBV was significantly correlated with ΔPV (*r*S = 0.842, *p* = 0.002) and ΔtHb-mass (*r*S = 0.782, *p* = 0.008), but not with ΔHb (*r*S = −0.188, *p* = 0.602).

## Discussion

The key finding of this study was that in elite rowers after a period of reduced training loads, changes in P_max_ were positively and directly associated with changes in PV and BV, but not with changes in tHb-mass and Hb.

Regardless of differences in testing methodology, mean values of maximal and submaximal power in the GXT (Table 3) were comparable in our rowers to Croatian (Mikulic, 2012) and German athletes (Treff et al., 2014b), representing the highest level of rowers in the world. However, in contrast to the Croatian data, in which seasonal variability (November–July) in mean absolute submaximal and maximal power was 14% and 6%, respectively, in our rowers the average changes in these variables (from July to January) did not exceed 2% despite a 34% reduction in training volume, although individual changes varied considerably (from −11.0 to 8.3%). The relatively high-power values in January may reflect the higher volume of training on the rowing ergometer prior to testing. Furthermore, some authors have reported (Klusiewicz, 1993) that restoration of submaximal power (at 4 mmol/l LA) in rowers after a period of reduced training loads is relatively fast and may occur early in the season after an increase in training volume to a moderate level. In contrast, greater seasonal variability was found in our rowers in rel.P_max_, which decreased on average by −4.0% (ranging from −14.2 to 3.0%) as their BM increased by 2.5% (ranging from −1.7 to 5.9%). This was accompanied by decreases in La_3_' (−16.3%, ranging from −31.4 to −4.8%) and HR_max_ (−2.4%, ranging from −7.0 to 0.5%).

Furthermore, tHb-mass and BV in our athletes (Table 3) were comparable to German rowers at the end of the preparation period of the annual training cycle, which averaged 13.7 ± 0.5 g/kg and 101 ± 6 ml/kg, respectively (Treff et al., 2014b). The lack of differences in the mean values of tHb-mass, both in absolute and relative terms, in our rowers (who were not iron deficient) is consistent with previous observations indicating stability in this variable during long training periods in high-level endurance athletes (Eastwood et al., 2008; Garvican et al., 2010; Prommer et al., 2008). However, similar to other authors who have investigated hematological adaptations to different types of training in high-level athletes (Hauser et al., 2016; Nummela et al., 2021), we found relatively large individual variability in ΔtHb-mass (ranging from −7.8 to 7.7%), also after a reduction in training volume. Even greater individual variability of ΔtHb-mass was observed by other authors (Eastwood et al., 2012) in recreational triathletes, where after 30 days of detraining, one athlete showed a decrease of 8%, while another showed an increase of about 12% in tHb-mass after 10 days only.

In our study, mean values of PV were related to training volume, being higher in the competitive phase and lower with a reduction in training volume (Table 3). This is consistent with the results of many studies demonstrating a positive association between training loads and PV (Astolfi et al., 2021; Sawka et al., 2000). However, because of differences in ambient temperature during the two measurement periods (minimum/maximum temperatures in the days preceding July and January testing were +17/+28°C and −5/−1°C, respectively), PV may also have been influenced by environmental factors. This idea is supported by the findings of other authors, who reported that endurance training in the warm summer months was associated with an increase in PV (Astolfi et al., 2021), and conversely, body exposure to cold stress was associated with a decrease in PV, caused mainly by a shift of water from the vascular to the interstitial space (Vogelaere et al., 1992).

Despite a decrease in PV, mean values of Hb remained stable in our rowers over the 6-month observational period, and ΔPV was not significantly correlated with ΔHb. Additionally, despite a decrease in mean PV (being part of the BV), mean BV did not change between the two evaluations (Table 3). The mechanism underlying this observation is not clear yet, but it may be at least partially explained by differences in the hematological responses to training performed in different parts of the season; BV expansion in summer was only due to PV expansion, but in winter, this occurred due to both plasma and cellular phases (Shapiro et al., 1981). Elucidation of this issue is beyond the aim of our study and requires further research. Nevertheless, it is worth noting that individual ΔBV in our rowers was positively and directly associated with ΔPV and ΔtHb-mass, but not with ΔHb.

A review of the scientific literature indicates that no studies have shown direct associations between changes in intravascular volumes or ΔtHb-mass and ΔEPP after a period of reduced training loads in elite athletes. Only weak correlations between ΔtHb-mass (*r* = 0.23) or ΔBV (*r* = 0.10) and ΔVO_2max_ were observed in recreational triathletes after a 30-day detraining period (Eastwood et al., 2012). In this study, factors other than hematological were considered to be responsible for the decrease in VO_2max_. A reduction in VO_2max_ (−6%) accompanying BV contraction (−9%) was observed in endurance-trained men after 2–4 weeks of detraining (Coyle et al., 1986). These variables returned to near trained levels when BV was expanded (by dextran infusion) to a level similar to that of trained subjects. Some authors (Bonne et al., 2014; Montero et al., 2015) also found that the elimination of hematological adaptations induced by endurance training in non-athletes was associated with the loss of post-training increases in VO_2peak_ and peak power in the GXT. In our Olympic rowers, ΔP_max_ after reduced training was very strongly correlated with ΔPV and ΔBV. This confirms the dominant role of hematological factors (central adaptations) for maximal exercise capacity in high-level endurance athletes. However, unlike non-athletes, acute PV expansion in elite endurance athletes, who already possess a high BV, does not improve their VO_2max_ and endurance performance (Warburton et al., 1999). A rather negative impact of the decrease in PV on P_max_ was noticeable in our rowers (Figure 1).

Associations between changes in submaximal power (at the lactate threshold) and changes in intravascular volumes or tHb-mass were weaker in our athletes and not statistically significant, which may suggest that changes in submaximal power relied more on nonhematological factors (e.g., skeletal muscle adaptations) than on hematological adaptations.

Our study had several strengths, but also some limitations. The study cohort consisted exclusively of elite athletes in an Olympic sport, who performed almost the same controlled training under the same environmental conditions and maintained a similar diet supervised by a sports dietitian. Furthermore, all our participants were regularly subjected to national and international doping control, both in- and out-of-competition; thus, we did not anticipate any issues related to misuse of banned substances. However, we were not able to determine the intensity of training other than by relying on the coach’s discretion. In addition, due to the differences in ambient temperature at the two measurement points (warm summer and cold winter), it cannot be ruled out that environmental factors had at least a partial effect on PV changes. Therefore, seasonal elements should be considered when evaluating the hematological effects of training.

## Conclusions

After a period of reduced training loads in elite rowers, changes in P_max_ were strongly correlated with changes in PV and BV, but not with changes in tHb-mass and Hb. These findings indicate a close relationship between changes in intravascular volumes and maximal exercise capacity in high-level endurance athletes.
